# Here comes the sun: music features of popular songs reflect prevailing weather conditions

**DOI:** 10.1098/rsos.221443

**Published:** 2023-05-03

**Authors:** Manuel Anglada-Tort, Harin Lee, Amanda E. Krause, Adrian C. North

**Affiliations:** ^1^ Faculty of Music, University of Oxford, Oxford, UK; ^2^ Computational Auditory Perception Group, Max Planck Institute for Empirical Aesthetics, Frankfurt am Main, Germany; ^3^ Max Planck Institute for Human Cognitive and Brain Sciences, Leipzig, Germany; ^4^ Department of Psychology, James Cook University, Townsville, Australia; ^5^ School of Population Health, Curtin University, Perth, Australia

**Keywords:** weather, seasons, mood, emotion, music preferences, media consumption

## Abstract

We examine associations between prevailing weather conditions and music features in all available songs that reached the United Kingdom weekly top charts throughout a 67-year period (1953–2019), comprising 23 859 unique entries. We found that music features reflecting high intensity and positive emotions were positively associated with daily temperatures and negatively associated with rainfall, whereas music features reflecting low intensity and negative emotions were not related to weather conditions. These results held true after controlling for the mediating effects of year (temporal patterns) and month (seasonal patterns). However, music–weather associations were more nuanced than previously assumed by linear models, becoming only meaningful in those months and seasons when changes in weather were the most notable. Importantly, the observed associations depended on the popularity of the music: while songs in the top 10 of the charts exhibited the strongest associations with weather, less popular songs showed no relationship. This suggests that a song's fit with prevailing weather may be a factor pushing a song into the top of the charts. Our work extends previous research on non-musical domains (e.g. finance, crime, mental health) by showing that large-scale population-level preferences for cultural phenomena (music) are also influenced by broad environmental factors that exist over long periods of time (weather) via mood-regulation mechanisms. We discuss these results in terms of the limited nature of correlational studies and cross-cultural generalizability.

## 1. Introduction

Music is central to the human experience and a ubiquitous element in everyday life for most people around the world [1,2]. In part, this is because music listening fulfils numerous psychological needs, one of the most important being mood regulation [3–6]. The ability of music to influence emotions characterizes practically every musical activity, from composition and performance, to music listening and the many applications of music in society, such as film, marketing and therapy. For example, people often choose to listen to music to change, release or match their current emotions (e.g. [7]). Importantly, the way in which people regulate their mood has an impact on their physical health, psychological well-being and cognitive functioning [8–10]. However, the majority of research concerning the social psychology of music listening has either considered factors internal to the individual, such as age and personality (e.g. [11–13]), or factors related to the context and social situation where the listening is taking place (e.g. [14–17]). Very little research on musical behaviour has considered broad environmental influences that exist over extended periods of time, such as seasonal patterns and prevailing weather conditions.

Two previous studies have investigated specifically the relationship between seasons and music preferences [18,19]. They found that when participants are primed or asked to think of certain seasons, their music preferences change systematically, including a greater preference for energetic music in spring and summer and ‘melancholic' music in winter. However, this research relies on self-report, hypothetical listening scenarios and a small number of participants. By contrast, the increasing availability of data from on-demand music streaming services makes it possible to study patterns of music consumption over longer periods of time across many individuals. Indeed, a large-scale study on music consumption in a sample of 1 million listeners found consistent diurnal and seasonal patterns in preferences for certain music features across 51 countries [20]. The results indicated that more energetic music was preferred during normal working hours, while more relaxing music was preferred late at night, suggesting that music choice shapes and reflects the listeners' mood.

Although the relationship between weather and music consumption remains unexplored, there is a body of research concerning associations between weather and various non-musical aspects of mood and behaviour. At the risk of over-generalizing, there is some evidence that sunshine and warmth are associated with greater activity and positivity in attitudes and behaviour, whereas colder weather is associated with less active and more reflective affect and behaviour. In the context of consumer and business psychology, several studies have argued that physical warmth increases the salience of psychological warmth and activity, with implications for various commercial attitudes and behaviours. For instance, some studies have reported that sunny weather is correlated positively with daily stock market returns [21,22]. Other studies have found that more exposure to sunlight decreases negative affect and increases consumer spending [23–26], whereas warm temperatures increase consumers' valuation of products [27]. Relationships between weather and attitudes and behaviour are not limited to business and consumer psychology. Criminological research indicates that daily temperature is associated with higher levels of aggravated crime [28] and property crime [29–33]; and arguably the best-known work in this space concerns the relationship between weather and mood disorders, most notably seasonal affective disorder, such that depressive symptoms are experienced more commonly in colder, darker months (e.g. [34–36]).

As this brief review indicates, music preferences are sensitive to seasonal patterns, and the nature of this appears to be related to mood-regulation mechanisms. Similarly, although the specific nature of the relationships often remains subject to empirical debate, there is a significant quantity of evidence indicating that mood and behaviour are related to weather so that more clement weather is associated with increased activity and positive affect. Here, we investigate the possibility that prevailing weather is related to the activity and valence of music that is popular at the time. We analysed key music features of all songs that reached the United Kingdom weekly top charts for a period of 67 years (1953–2019), comprising more than 20 000 unique songs. High-level audio features were estimated using music information retrieval techniques that rely on state-of-the-art machine learning models trained on large music corpora. We compared the resulting dataset of popular music features with monthly weather variables (temperature, sunshine, rainfall) collected during the same time span. Moreover, we examined the extent to which music–weather associations were determined by the position of the songs in the charts. This was motivated by the idea that hyper popular hits may be better at reflecting consumers' preferences and prevailing environmental conditions. We examined this possibility by comparing weather–music associations between songs that reached the top 10 positions in the charts and songs in chart positions 91–100.

## 2. Methods

### 2.1. Weather variables

Weather data was gathered from the Meteorological Office of the United Kingdom (https://www.metoffice.gov.uk/), which provides time-series data from the United Kingdom at the month level. We collected weather data on three key metrics, namely monthly mean of daily maximum air temperature (in degrees Celsius), monthly total duration of bright sunshine (in hours), and days in the month with precipitation amount greater than 1 mm. To match the availability of the music features dataset (see below), weather variables were collected from 1953 to 2019.

### 2.2. Music features

We collected data on high-level music features (i.e. psychological measures that can be estimated from raw audio recordings) of all available songs that reached the top 100 weekly charts of the United Kingdom since the beginning of the official singles chart (https://www.officialcharts.com/) in November 1952 [37] to the year this research was conducted (2020). We only included years where we had data for the full 12 months, resulting in a total of 67 years from 1953 to 2019.

Music features were collected from Spotify's database using the following procedure: for each song in the dataset, a query containing its title and artist name was sent to Spotify's database using the application programming interface (API), retrieving up to 50 search results per call. To find the closest match (e.g. there are multiple versions of the same song in the Spotify database, including remastered versions or radio edits) and to allow for any spelling errors, we computed string similarity scores using the q-gram method in ‘stringdist' [38]. Q-gram was calculated by taking the sum of absolute differences between n-gram vectors of two strings. Through extensive piloting, we found that n-gram of size 3 and a similarity score higher than 0.70 (after normalizing the values to range between 0 and 1) was adequate for obtaining robust matches. Consequently, among the 50 searches per song, the one with the highest string similarity score was chosen. If there were no searches that reached a score of 0.70, we treated the song as unfound.

Next, we sent another API query to retrieve all the information related to music features for the found songs. Spotify's features have been widely used in numerous studies to measure perceptual and psychological dimensions of large music datasets (e.g. [20,39]). Moreover, a recent study comparing Spotify's mood estimates with cross-cultural participants confirmed the robustness of the algorithm to measure affective features of popular music [40]. Here, we only considered high-level features representing psychological dimensions, comprising a total of nine music features^1^:

— *Acousticness*. The likelihood that the track was recorded only by acoustic instruments (0–1 scale).
— *Danceability*. How suitable the track is for dancing based on a combination of musical elements, such as tempo and rhythm (0–1 scale).
— *Energy*. A perceptual measure of intensity and activity (0–1 scale).
— *Instrumentalness*. The likelihood that the track is predominantly instrumental (0–1 scale).
— *Liveness*. The extent to which a live audience can be detected in the track (0–1 scale).
— *Loudness*. The subjective perception of sound pressure (decibels).
— *Speechiness*. The extent to which words are detected in the track (0–1 scale).
— *Tempo*. The overall estimated tempo (speed or pace) of a track (beats per minute).
— *Valence*. How the track is emotionally perceived, from negative to positive emotions (0–1 scale).

We obtained available data for 23 859 unique songs from 7398 unique artists. To allow comparison between the music data (collected at the week level) and the weather data (collected at the month level), we aggregated each of the Spotify variables at the month level by taking the mean.

## 3. Results

**Figure 1.**
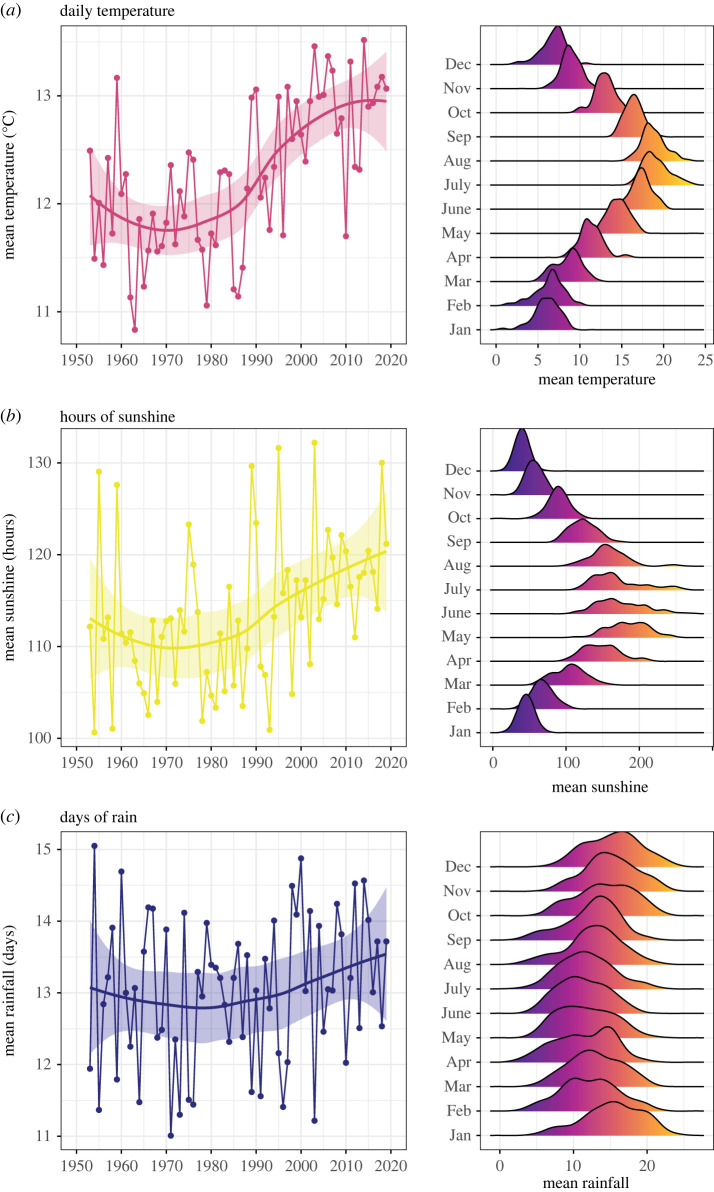
Weather variables used in the study (summarized at the year and month level). (*a*) Mean daily maximum air temperature (in degrees Celsius). (*b*) Total duration of bright sunshine (in hours). (*c*) Days in the month with precipitation amounts greater than 1 mm. Note: Each point in the left column plots is a yearly average and the smooth line was estimated with local polynomial regression fitting (loess). Distributions in the right column plots were estimated for each month collapsing over the 67 years and using kernel smoothing.

Figure 1 summarizes the three weather variables (i.e. daily temperature, hours of sunshine and days of rain) at the year and month levels. Values for the three weather variables increased slightly over the years and varied systematically over the months, following the expected seasonal patterns in the United Kingdom. Thus, trends in weather and some of the music features are confounded by sharing common year-level dynamics. For example, popular music has become more danceable over the years, presumably as a result of the birth of electronic music [41]. To remove this confound, we normalized both weather and music variables using *z*-scores computed independently for each year. Consequently, the transformed data had the same baseline across all years, effectively removing confounded year-level dynamics.

When computing intercorrelation between the music features extracted from Spotify, we identified that some variables were highly correlated with one another. Thus, we performed a principal component analysis (PCA) to reduce the dimensions and have more interpretable results. The PCA identified a two-component solution that explained 56.25% of the variance (see appendix A for details on the PCA and the individual contributions of each audio feature). The first PCA component explained 37.5% of the variance in the correlation matrix and corresponded to positive valence and high intensity (henceforth referred to as *high-arousal positive music* factor). This component is very similar to the musical intensity factor found by Park *et al*. [20], with high scores reflecting energetic music (e.g. danceable, loud and fast tempo) with positive valence (e.g. happy, cheerful, euphoric emotions). The second PCA component explained 18.7% of the variance and corresponded to negative valence and low intensity (henceforth referred to as *low-arousal negative music* factor). High scores on this second component reflect music with negative valence (e.g. sad, depressed, angry emotions) that is not suitable for dancing and has less vocal presence.

### 3.1. Music–weather associations

Much of the existing literature has assumed that the relationship between weather and behaviour is linear, so that increases in sunshine and temperature are incrementally ‘positive'. While this may often be correct, it may not always be the case. For example, very high temperatures could be perceived negatively, or a sunny day in cold winter months may have a much positive impact on mood than the same amount of sun during summer. Thus, we used generalized additive models (GAMs) to consider nonlinear associations [42,43] between music features and weather conditions. A GAM divides the full range of the predictor variable (e.g. hours of sunshine) into smaller parts and assigns an individual function to each part in order to fit the data. A spline or smooth function is then built out of the smaller individual functions.

We ran a separate GAM^2^ for each of the two principal components found in the PCA as the dependent variables, and a factor-smooth interaction to fit different smooths for each weather measurement as the predictors (temperature, sunshine and days of rain). We used two complementary approaches to test the significance of each smooth term. First, we looked at *p*-values for smooth components using a Wald-type test to indicate the likelihood that the splines that make up the function are jointly zero [43]. Second, we compared the smooth term with a 95% confidence interval (CI) in each plot with a straight line with an intercept at 0. A non-significant smooth term is one where a horizontal line can be drawn through the 95% CI.

**Figure 2.**
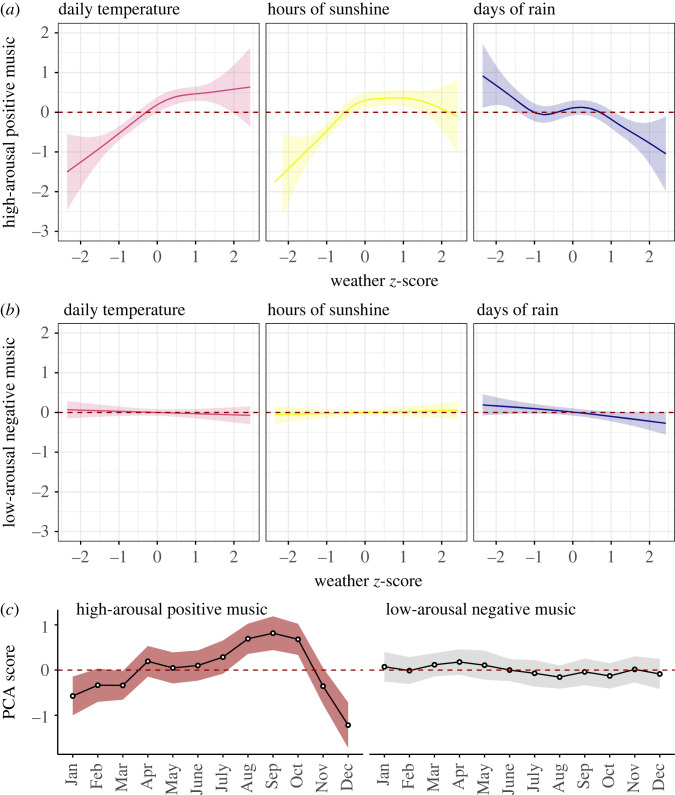
Associations between music features and prevailing weather conditions. (*a*) GAM plots showing the relationship between weather conditions and features reflecting high-arousal positive music and (*b*) features reflecting low-arousal negative music. (*c*) Seasonal patterns (monthly mean with 95% CI) in the two PCA components. Note: The red dashed line in all plots is set to intercept = 0 to indicate null effects; shaded areas indicate 95% CI.

Figure 2 shows the results of the two GAMs. The GAM concerning high-arousal positive music (figure 2*a*) converged after six iterations and explained 6.09% of the variance (adjusted-*R*^2^). Both mean daily temperature (*F*_2.53,3.17_ = 23.70, *p* < 0.001) and hours of sunshine (*F*_3.26,4.07_ = 14.89, *p* < 0.001) showed a significant positive curvilinear relationship. However, the strength of the association was strongest when daily temperature and sunshine hours were low (less than 0 *z*-score), suggesting that these weather variables are more influential in months when they are scarce (e.g. months with low temperatures and a few hours of sunshine). By contrast, the mean number of days with rain was negatively associated with high-arousal positive music (*F*_3.87,4.83_ = 4.85, *p* < 0.001). This relationship was also curvilinear, showing the strongest associations when days of rain were either the lowest or the highest (in months either with very few or many days of rain on average).

The GAM concerning low-arousal negative music converged after nine iterations and explained 0.07% of the variance. As shown in figure 2*b*, none of the smooth terms were significantly associated with negative and calm music (all *p*-values > 0.05).

Figure 2*c* shows the role of seasonal patterns on the two PCA music factors, plotting the average (and 95% CIs) across each month. The results indicated a significant influence of month on high-arousal positive music, with scores statistically above 0 (null effect) in warmer months (July to October) and below 0 in colder months (November to February). By contrast, features reflecting low-arousal negative music remained mostly flat (around 0) over the months, indicating no effect of seasonal patterns.

While seasonal patterns are important in understanding associations between weather conditions and music preferences, there are various factors that change from season to season other than weather conditions (e.g. people celebrate Christmas in winter, tend to travel in summer, etc.). Thus, we performed two complementary control analyses to study whether the relationship between weather and high-arousal positive music remained significant after controlling for seasonal effects. First, we ran a GAM for each weather variable separately (temperature, sunshine, rainfall) but this time included season as a covariate in the model. Specifically, season was modelled by adding a factor-smooth interaction between the weather variable and each season—i.e. winter (December to February), spring (March to May), summer (June to August) and autumn (September to November). This model, therefore, controls for seasonal effects by adding season as a covariate.

The GAM with daily temperature explained 9.96% of the variance (adjusted-*R*^2^) and revealed that weather–music associations only remained significant in autumn (*F*_2.299,2.850_ = 9.41, *p* < 0.001), where higher temperatures were positively associated with music containing high-arousal positive features (weather–music associations in the other seasons were non-significant, all *p*-values > 0.05). The GAM with hours of sunshine explained 9.79% of the variance and showed that weather–music associations remained significant in autumn (*F*_2.350,2.933_ = 6.70, *p* < 0.001) and spring (*F*_1.001,1.003_ = 5.72, *p* = 0.02), where more hours of sunshine related positively with high-arousal positive music. Finally, the GAM with days of rain explained 10.5% of the variance and indicated that weather–music associations remained significant in autumn (*F*_1.0,1.001_ = 8.79, *p* = 0.003) and winter (*F*_4.72,5.82_ = 3.69, *p* = 0001), where more days of rain were negatively associated with high-arousal positive music. Together, these results show that associations between prevailing weather conditions and high-arousal positive music remained significant when controlling for seasonal patterns only in some of the seasons (consistently in autumn for all weather variables and also in spring and winter for sunshine hours and days of rain, respectively).

In a second control analysis, we used a stricter approach by normalizing all weather variables and the music factor within each month using *z*-scores. Thus, the transformed data had the same baseline values across all months and years, effectively removing confounding effects of longitudinal trends (differences between years) and season (differences between months). We then repeated the same GAM approach described in the main analysis (figure 2*a*). The results of the GAM showed that removing monthly patterns from the data significantly decreased the strength of weather–music associations (adjusted-*R*^2^ = 0.005). However, mean daily temperature (*F*_1.00,1.01_ = 4.27, *p* = 0.04) and days of rain (*F*_3.59,4.52_ = 2.78, *p* = 0.02) remained significant predictors of high-arousal positive music (following similar trends to those observed in the previous analysis), whereas hours of sunshine turned non-significant (*p* > 0.05).

### 3.2. Song popularity

Lastly, we examined the extent to which the significant associations between weather and high-arousal positive music depended on the popularity of the songs. In particular, we compared the music–weather associations within the weekly top 10 most popular songs in the charts against the same associations in songs that were comparatively less popular (the bottom 10 songs, with rank positions 91–100). For this analysis we only included years for which data on the weekly top 100 songs was available, beginning in 1994 and comprising a period of 25 years until 2019. For each month, we sub-grouped the songs that entered 1–10 rank positions (top 10) and 91–100 rank positions (bottom 10) in the chart. We repeated the same pipeline used above to curate the dataset (i.e. averaging variables on a monthly basis, scaling at the year level and running a PCA with all music features). The PCA derived very similar results to the earlier analysis of the entire dataset, namely a two-component solution that explained 57.66% of the variance, where the first component (37.10%) corresponded to high-arousal positive music and the second component (20.56%) corresponded to low-arousal positive music (see appendix B for details on the PCA and the individual contributions of each audio feature).

**Figure 3.**
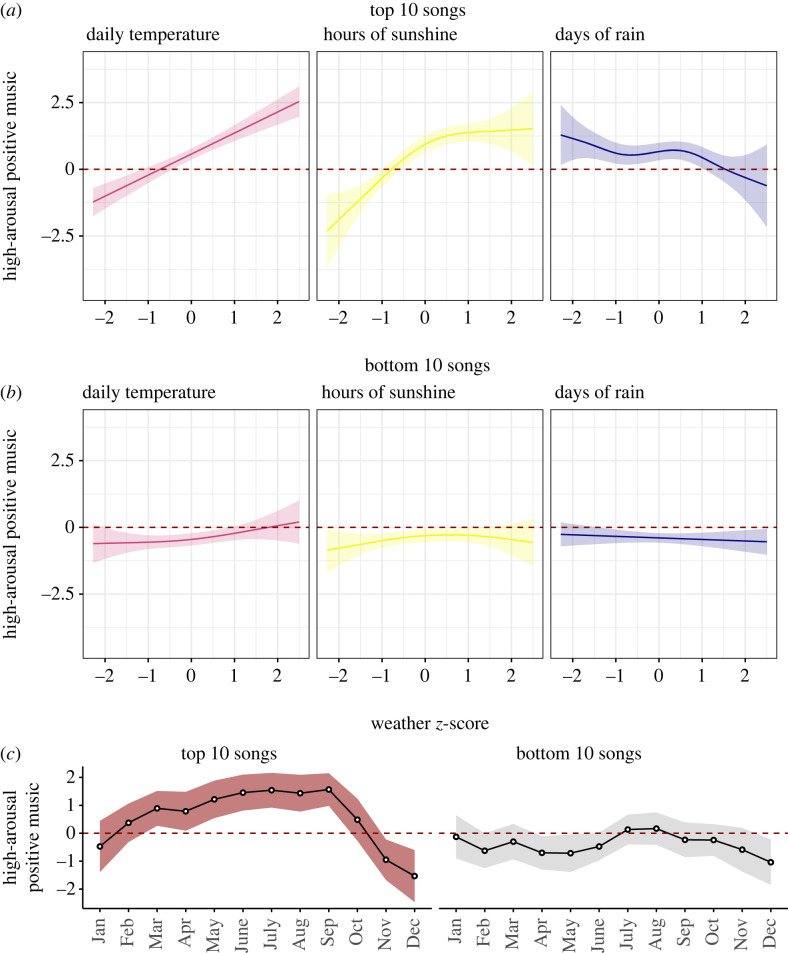
Weather–music associations in the top 10 (rank positions 1–10) and bottom 10 (rank positions 91–100) songs of the charts. GAM plots showing the relationship between weather conditions and high-arousal positive music features for those songs that ranked on the (*a*) top 10 and (*b*) bottom 10 of the charts. (*c*) Seasonal patterns (monthly mean with 95% CI) for high-arousal positive features of songs at the top versus the bottom of the charts. Note: The red dashed line in all plots is set to intercept = 0 to indicate null effects; shaded areas indicate 95% CI.

Figure 3*a* shows the results of the GAM using the top 10 most popular songs and the high-arousal positive music factor, which explained 12.20% of the variance (adjusted-*R*^2^). We obtained a similar pattern of results to those found in the main analyses (figure 2*a*), revealing significant associations between features reflecting high-arousal positive music and daily temperature and hours of sunshine (all *p*-values < 0.001). However, the negative trend between music features and days of rain was no longer significant (*p* = 0.09). Figure 3*b* shows the results of the same analysis using the bottom 10 songs. This time the GAM showed that none of the weather variables were associated with high-arousal positive music (all *p*-values > 0.05; adj-*R*^2^ = 0.002). Moreover, figure 3*c* shows that features reflecting high-arousal positive music in the top 10 songs were significantly influenced by seasonal patterns, whereas the same features in the bottom 10 songs remained comparatively flat over the months.

## 4. Discussion

While previous research in non-musical domains (e.g. consumer behaviour, crime, mental health) has shown that weather conditions are associated with certain moods and behaviours, the relationship between weather and music consumption remains hypothetical. This study addressed this gap by examining the relationship between prevailing weather conditions and music features of all songs that reached the United Kingdom weekly top charts over 67 years (1953–2019). Our results showed that variations in weather conditions are associated with music features reflecting positive valence and high intensity, whereas features reflecting negative valence and low intensity were not related to weather at all (figure 2*a*,*b*). We found similar results when looking at seasonal patterns: the prevalence of high-arousal positive music fluctuated following the expected seasonal trends in the United Kingdom (with the highest values in summer and the lowest in winter), whereas the amount of low-arousal negative music remained unchanged throughout the months (figure 2*c*).

Our results are consistent with previous studies showing associations between music preferences and seasons in hypothetical listening scenarios [18,19], and with large-scale quantitative data showing that music features reflecting intense and positive music vary consistently according to seasonal patterns across cultures [20]. However, our findings extend previous research by showing that large-scale music consumption patterns may also be linked to prevailing weather conditions via mood-regulation mechanisms. More broadly, our results add to the relatively small body of research identifying social influences on music preferences at a broad cultural level, such as macroeconomic factors (e.g. [45–47]), psychological and cultural values (e.g. [48–50]) and social preferences and norms [16].

Importantly, we show that not all combinations of features in popular music relate to weather—i.e. we only found significant weather associations with music features reflecting high-arousing positive music. That is, songs that are danceable, energetic, loud, fast and evoke positive emotions such as happiness, joy and activeness. Songs scoring high in this music factor include ‘Ice Ice Baby' by the Glee Cast, ‘Temperature' by Seaon Paul and ‘Get Loose' by Evelyn King. Note that low values in this music factor indicate the absence of negative feelings, not their presence. It is unclear, however, why music features reflecting low intensity and negative valence (e.g. evoking emotions such as sadness or anger) did not relate to variations in weather. One possibility is that negative affect states are more idiosyncratic and, consequently, influenced by individual situational factors rather than general environmental conditions.

To examine weather–music associations we used GAMs [42,43], a flexible method for examining complex and nonlinear relationships. This method revealed that music–weather associations are more nuanced than previously assumed by linear models. For example, we found that the relationships between high-arousal positive music and both daily temperature and hours of sunshine were curvilinear: music–weather associations were significant only when temperature and hours of sunshine were low, but when they reached average levels, the relationships ceased to be significant. This suggests that daily temperature and hours of sunshine are only influential in months when the weather is generally bad, such as months with low daily temperatures and hours of sunshine (e.g. winter, autumn). By contrast, days of rain were negatively associated with high-arousing positive music, also in a nonlinear fashion. That is, the negative association was only significant in months with extreme values of rainfall (either very dry or very wet months). Again, this suggests that weather conditions only reflect changes in mood in those months when weather changes are notably different. In fact, a control analysis (using season as a covariate) indicated that associations between weather and high-arousal positive music were only significant in some of the seasons. This further supports the finding that dynamics in weather conditions are most influential in seasons when changes are most notable (e.g. autumn). For example, the impact of a sunny month in autumn may be larger than the impact of a sunny month in summer.

If people often choose music to regulate their emotions [7], and popular music is determined by consumer choices, it is possible that hyper popular hits are better at reflecting changes in weather than less successful songs. Indeed, we found significant weather–music associations when looking only at songs that reached the top 10 positions in the charts. By contrast, these associations disappeared when looking at less popular songs that charted in positions 91–100 (figure 3*a*,*b*). A similar pattern of results occurred when looking at seasonal trends: the musical features of hyper popular songs fluctuated according to seasons, whereas the features of less successful songs were unrelated to changes in season (figure 3*c*). This finding is consistent with previous research showing that the most successful songs in the charts exhibit distinct dynamics compared with less popular music [41]. In particular, it shows that hyper popular hits are better at reflecting environmental changes such as seasons and weather, suggesting that the fit of a song with prevailing environmental conditions may be a factor contributing to its success in the charts.

Naturally, this is a correlational study so the results must be interpreted with caution. However, we took several precautions to address this limitation. First, in all analyses we removed confounding trends over time by normalizing all variables within each year, effectively removing yearly differences over time. Second, we performed a control analysis by adding season as a covariate in the model, revealing that after controlling for the effect of season, music–weather associations remained significant in some seasons (e.g. autumn, spring, winter). Finally, we performed a stricter control analysis using the same analysis strategy but removing any differences between months by normalizing all variables at the month level. After removing monthly and seasonal trends from the data, the associations between high-arousal positive music and both daily temperature and days of rain remained statistically significant, although the strength of the relationship decreased substantially.

Furthermore, alternative explanations may account for the results reported here. We emphasize the role of commercial factors. For example, for a piece of music to become featured for radio airplay, it must first satisfy a number of musical and non-musical criteria determined by industry gatekeepers. The advent of music streaming services may over time dilute the role of radio airplay in commercial success by providing access to a much wider range of music, but it comes with a new set of selection biases, such as those introduced by recommendation algorithms [51,52]. Thus, while weather conditions may relate to music consumption, it is also possible that music industry gatekeepers, or more recently recommendation algorithms, significantly bias the content of the music to which the public is exposed regularly. Finally, our analysis is limited to the United Kingdom. It is not clear whether the findings would generalize to very hot or dry climates, where high temperatures and high hours of sunshine may not be perceived positively and rainfall is perhaps more likely to be regarded positively. We see great potential for cross-national studies comparing a diverse sample of countries with different climate patterns and cultural differences in subjective well-being (e.g. [53]).

In conclusion, our results are consistent with the notion that prevailing weather conditions are associated with the popularity of music that is energetic and evokes positive emotions, at least in the United Kingdom. However, we found that music–weather associations are more nuanced than previously assumed, becoming meaningful only in those months and seasons when changes in weather are the most notable, such as winter and autumn. Moreover, the strength of this relationship depends on the popularity status of the music. These results contribute to the existing literature by suggesting that large-scale population-level preferences for cultural phenomena, such as music, are influenced by broad environmental factors that exist over extended periods of time via mood-regulation mechanisms.

## Figures and Tables

**Figure 4.**
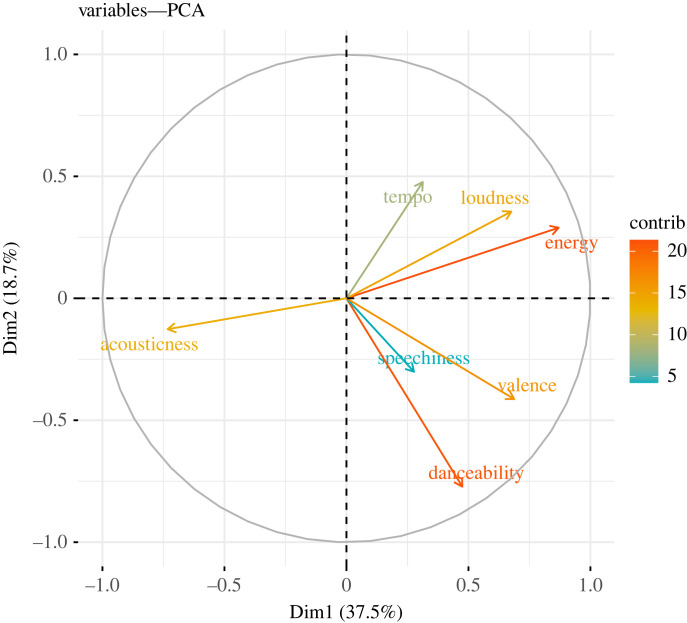
Principal component analysis on music features of all songs.

**Figure 5.**
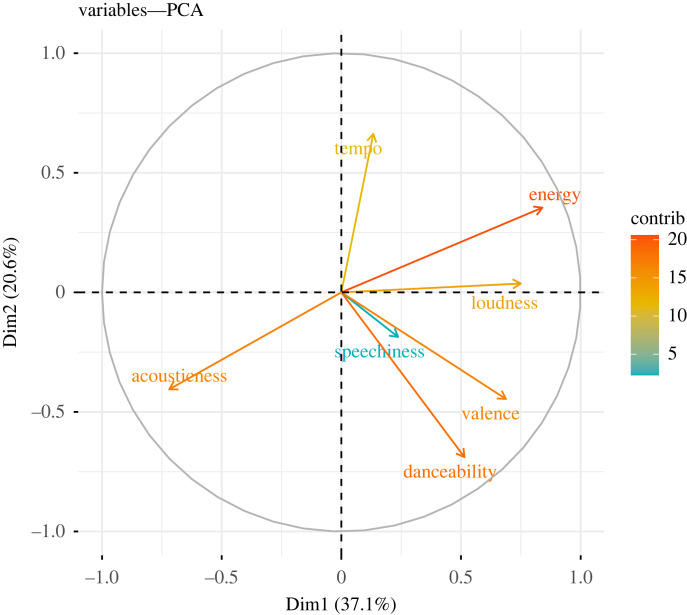
Principal component analysis on music features of top 10 and bottom 10 songs.

## Data Availability

All datasets and analysis code supporting this paper are stored in GitHub: https://github.com/manuelangladatort/music-and-weather and have been archived within the Zenodo repository: https://zenodo.org/badge/latestdoi/562985935 [54].

## Footnotes

1 See Spotify's Web API Developer Guide for full definitions; https://developer.spotify.com/documentation/web-api.

2 GAMs were implemented using the R package *mgcv* [44], assuming a Gaussian distribution of the errors and using the default method to select smoothing parameters, i.e. restricted maximum likelihood (REML). The residuals for each smooth term were randomly distributed (all *p*-values > 0.05) and their concurvity was low (all values less than 0.29).

## Appendix A

The audio features liveness and instrumentalness were removed because their Kaiser–Meyer–Olkin (KMO) values were lower than 0.5. We ran a PCA on the resulting audio features (acousticness, danceability, energy, loudness, speechiness, tempo and valence). Bartlett's test (*p* < 0.001) and the Kaiser–Meyer–Olkin (KMO = 0.61) measure verified the sampling adequacy for the analysis (all KMO values for the individual features were above 0.5). The scree plot indicated a solution with two factors, and there were only two PCA components with an eigenvalue larger than 1, which explained 56.25% of the variance. Thus, we accepted this two-component solution and computed the corresponding PCA scores for each component (figure 4).

## Appendix B

We ran a PCA on the same audio features used in the main analysis (acousticness, danceability, energy, loudness, speechiness, tempo and valence). Bartlett's test (*p* < 0.001) and the Kaiser–Meyer–Olkin (KMO = 0.63) measure verified the sampling adequacy for the analysis (all KMO values for the individual features were above 0.5). The scree plot indicated a solution with two factors and there were only two PCA components with an eigenvalue larger than 1, which explained 57.66% of the variance. Thus, we accepted this two-component solution and computed the corresponding PCA scores for each component (figure 5).
